# Identification and Validation of Metabolic Markers for Adulteration Detection of Edible Oils Using Metabolic Networks

**DOI:** 10.3390/metabo10030085

**Published:** 2020-02-29

**Authors:** Xinjing Dou, Liangxiao Zhang, Xiao Wang, Ruinan Yang, Xuefang Wang, Fei Ma, Li Yu, Jin Mao, Hui Li, Xiupin Wang, Peiwu Li

**Affiliations:** 1 Oil Crops Research Institute, Chinese Academy of Agricultural Sciences, Wuhan 430062, China; douxj521@163.com (X.D.); wangxiao0613@163.com (X.W.); yanrinannan@126.com (R.Y.); wangxuefang01@caas.cn (X.W.); mafei01@caas.cn (F.M.); yuli01@caas.cn (L.Y.); maojin106@whu.edu.cn (J.M.); lihui-gf@163.com (H.L.); wangxiupin@caas.cn (X.W.); peiwuli@oilcrops.cn (P.L.); 2 Key Laboratory of Biology and Genetic Improvement of Oil Crops, Ministry of Agriculture and Rural Affairs, Wuhan 430062, China; 3 Laboratory of Quality and Safety Risk Assessment for Oilseed Products (Wuhan), Ministry of Agriculture and Rural Affairs, Wuhan 430062, China; 4 Quality Inspection and Test Center for Oilseed Products, Ministry of Agriculture and Rural Affairs, Wuhan 430062, China; 5 Key Laboratory of Detection for Mycotoxins, Ministry of Agriculture and Rural Affairs, Wuhan 430062, China

**Keywords:** metabolic markers, edible oil, adulteration detection, metabolic network, LC-MS/MS

## Abstract

Food adulteration is a challenge faced by consumers and researchers. Due to DNA fragmentation during oil processing, it is necessary to discover metabolic markers alternative to DNA for adulteration detection of edible oils. However, the contents of metabolic markers vary in response to various factors, such as plant species, varieties, geographical origin, climate, and cultivation measures. Thus, it is difficult to identify a universal marker for all adulterants that may be present in some authentic samples. Currently, the specificity and selectivity of metabolic biomarkers are difficult to validate. Therefore, this study developed a screening strategy based on plant metabolic networks by developing a targeted analytical method for 56 metabolites in a metabolic network, using liquid/liquid extraction–liquid chromatography–tandem mass spectrometry (LC-MS/MS). We identified a chain of 11 metabolites that were related to isoflavonoid biosynthesis, which were detected in soybean oils but not rapeseed oils. Through multiple-marker mutual validation, these metabolites can be used as species-specific universal markers to differentiate soybean oil from rapeseed oil. Moreover, this method provides a model for screening characteristic markers of other edible vegetable oils and foods.

## 1. Introduction

Food adulteration, a violation of consumer rights, is a serious problem that has attracted the attention of consumers and authorities. According to published data, olive oil, milk, honey, and saffron are the most common targets of adulteration [1]. Many researchers have focused on the identification of adulterants in foods, including meat [2], fish and seafood [3], edible oils [4], dairy products [5], and honey [6] over the past decades. Various methods were developed for food fraud and adulteration detection, including sensory evaluation [7], polymerase chain reaction (PCR) [8], and chromatographic and spectroscopic methods [9,10,11,12]. Spectroscopic methods such as infrared (near-infrared and Fourier-transform infrared FTIR) [13,14], synchronous fluorescence [15], Raman spectroscopy [16], nuclear magnetic resonance spectroscopy [17], ion-mobility spectrometry (IMS) [6,18,19], and electronic nose [20] coupled with chemometric analysis have been employed for authentication or detection of adulteration. These methods are rapid, low-cost, environmentally safe, and non-destructive. Metabolomics or metabolic profile-based detection methods, analyzing triacylglycerol [21], fatty acids [22,23,24], phytosterols [25], and volatile organic compounds (VOCs) [26], have been developed to build an adulteration detection model with the help of chemometric methods. The accuracy of the chemometric method depends on the number of training samples. Compared with the spectroscopic and metabolomic methods, detection methods based on the identification of DNA and metabolic markers could provide more confirmative results.

DNA hybridization and PCR are powerful molecular techniques that are used in food fraud detection [8]. Multiplex PCR has also been employed to simultaneously detect chicken, duck, and goose DNA in meat products derived from beef, pork, mutton, or quail. The method successfully identified the origin of animal species in manufactured meat products [27]. However, DNA markers are less accurate in discriminating cooked or highly processed foods such as refined edible oils. In fact, degumming is an important step in oil refining, which removes DNA from crude vegetable oil [28]. Thus, these processing steps make it difficult to perform accurate DNA analyses. As DNA markers could be influenced by certain treatments, it is necessary to discover alternative metabolic markers that are exclusive to the adulterant.

Only a few metabolic markers were identified for the detection of edible oil adulteration. For example, 4, 4′-dimethylsterols and filbertone are used as markers to detect hazelnut oil in virgin olive oils [29,30]; ∆7-stigmastenol is used as a marker to detect sunflower or soybean oil in olive oil [31], and brassicasterol is used as a marker for rapeseed oil [32]. Recently, a non-targeted metabolomics approach was proposed to identify food markers by discriminating organic from conventional tomatoes [33] as well as to determine the origin of extra-virgin olive oil [34]. However, due to differences in plant varieties, agricultural environments, and processing technologies, the content of potential metabolic markers varies significantly. Therefore, one marker detected in some cheap oils may not be universally present in all samples of this type of cheap oil. The detection methods using these markers usually suffer from high rates of false-negative results or false-positive results. Thus, screening specific and universal markers of adulterants is essential. Hence, if multiple metabolites in the same metabolic pathway are simultaneously detected in adulterant oil, we could infer that these metabolic markers are related to genetic factors of that oil species.

Based on its very reasonable price, soybean oil is one of the most common edible oils in the market. Therefore, it is often employed as an adulterant in more expensive edible oils, such as sesame oil, camellia oil, peanut oil, and rapeseed oil in China. Generally, isoflavones including genistein, genistin, daidzein, and daidzin are used as markers of soybean oil [35,36]. However, it is difficult to ensure they are not present in other edible oils. In this study, taking soybean oil (cheap) and rapeseed oil (expensive) as an example, the analysis of 56 metabolites in the same metabolic network was performed to identify and validate markers of soybean oil. 

## 2. Materials and Methods

### 2.1. Chemicals and Materials

Ultra-pure water (18 mΩ) was obtained from a Milli-Q water purification system (Millipore Co., Ltd., Milford, MA, USA). High-performance liquid chromatography (HPLC)-grade methanol, acetic acid, and 56 analytical standards, including catechinic, scopolin, chlorogenic acid, epicatechinic, vanillic acid, caffeic acid, purerarin, syringic acid, daidzin, glycitin, scopoletin, eriocitrin, umbelliferone, p-coumaric acid, dihydroquercetin, sinapic acid, genistin, liquiritin, ferulic acid, salicylic acid, rutin, isoferulic acid, m-coumaric acid, naringin, hesperidin, resveratrol, xanthotoxol, silydianin, sinapyl alcohol, o-coumaric acid, liquiritigenin, kaempferol, 2’-hydroxygenistein, eriodictyol, daidzein, psoralen, glycitein, quercetin, didymin, bergaptol, naringenin, luteolin, cinnamic_acid, hesperetin, genistein, bergapten, diosmetin, isoliquiritigenin, coumestrol, sinensetin, formononetin, medicarpin, imperatorin, biochanin A, tangeretin, and rotenone (displayed in Table S2), were purchased from Sigma-Aldrich Co., Ltd. The stock solutions of these authentic standards were 10.0 mg of each standard dissolved in 10 mL methanol. Then, the stock solutions were diluted to various concentrations before analysis. All stock solutions were sealed with Parafilm^®^ and stored in a −20 °C freezer.

### 2.2. Sample Preparation

To ensure the authenticity and reliability of the soybean samples, about half of the samples were prepared in the laboratory, whereas the other half were purchased from the market. For the soybean oils prepared by the laboratory, the oil was made from collected soybean seeds by using an oil presser in hot-pressing mode; the exudate was collected in a centrifuge tube (50 mL) that was later centrifuged at 8000× *g* for 5 min; the supernatant was transferred into an amber bottle, sealed with Parafilm^®^ and stored in the dark before use. The rapeseed oils prepared by the laboratory were obtained from Sinograin Corporation (Wuhan, China). There was a total of 17 rapeseed oils (11 commercial rapeseed oils (CRO) and 6 rapeseed oils prepared in another laboratory) and 18 soybean oils (9 commercial soybean oils (CSO) and 9 soybean oils prepared in our laboratory) used in the experiment. The detailed information of commercial oil samples was displayed in Table S1. 

Potential markers were extracted via liquid/liquid microextraction (LLME). In a previous study [37], the extraction methods of phenolic compounds in virgin olive oil, including solid-phase extraction (SPE), LLME, and ultrasound extraction (USE), were compared. The LLME method used in this study was originally obtained from the above literature and included a slight modification. LLME was performed by combining 6.00 g of each oil sample with 6 mL of H_2_O/MeOH (1:4, *v/v*); the mixture was extracted by vortex-mixing for 1.5 h; the extract was centrifuged at 15,000× *g* for 10 min at room temperature; 5 mL of the supernatant was transferred into another centrifuge tube and evaporated to dryness under a gentle stream of nitrogen gas; the residue was dissolved in 0.5 mL methanol with vortex-mixing, and the final extracts were filtered through a 0.22 μm organic filter and transferred into autosampler vials before injection into the LC-MS system.

### 2.3. UPLC-MS/MS Analysis of the Target Compounds

The quantification of the target compounds was achieved by UPLC (Finnigan, Waltham, MA, USA) coupled with a Thermo TSQ Quantum Ultra EMR mass spectrometer (Thermo Fisher Scientific, Waltham, MA, USA). A heated electrospray ionization (HESI) source was selected as the ion source. Processing of the spectral data and qualitative and quantitative analyses were performed using Xcalibur software version 2.0.7 (Thermo Fisher Scientific).

A C18 chromatographic column (Hypersil Gold, 100 mm × 2.1 mm i.d., 3 µm, Thermo Fisher Scientific) was selected for the separation of the target compounds. The column was maintained at 30 °C, and the injection volume was 3 μL. Methanol (A) and ultra-pure water with acetic acid (0.05%, *v/v*, B) were used as mobile phases. To increase the ionization efficiency of positive ions and to prevent the suppression by negative ions, acetic acid (0.05%, *v/v*) was added into the ultra-pure water. The flow rate was 200 μL/min, and a gradient elution program was applied as follows: 0–2 min 90% A; 2–2.5 min 90%–70% A; 2.5–5 min 70%–55% A; 5–8 min 55%–30% A; 8–13 min 30%–15% A; 13–13.5 min 15%–90% A, and holding for 2.5 min to equilibrate the column before the next injection.

Selected reaction monitoring (SRM) mode, offering high sensitivity as well as ability to perform qualitative and quantitative analyses, was used in this study. The parameters of the ion source were as follows: vaporizer temperature of 300 °C, capillary temperature of 320 °C, sheath gas pressure (N2) of 35 psi, auxiliary gas pressure (N2) of 5 arb, and collision gas (Ar) pressure of 1.5 mTorr. The spray voltage was +4000 V in the positive mode and -3,000 V in the negative mode. The cycle time was 2.0 s. The standard solutions (1 μg/mL in methanol) were directly injected by using a syringe pump to optimize parameters such as ionization mode, monitoring precursor ions, and lens voltage and collision energy of the product ions.

### 2.4. Data Processing and Statistical Analysis of Metabolites

Peak identification requires the accurate qualitative analysis of target compounds [38]. The peaks that appeared in the sample analysis were identified by comparison to the retention time (RT) and ion pairs of authentic standards. The identification criteria were as follows: RT within 0.2 min of the average calibrator RT, the presence of two transition ions, and the difference in relative ion intensities (% of base peak) within 20% [39]. Quantification was achieved by using an external standard. A standard solution with different concentrations was prepared by diluting the stock solution with methanol. Then, the standard curve was calculated from the relationship between the concentration and the peak area using standard solutions at different concentrations. Data acquisition and processing were conducted via Xcalibur software version 2.0.7 (Thermo Fisher Scientific). The limit of quantitation (LOQ) was calculated as the concentration of the analyte at a signal-to-noise ratio (S/N) of 10. The results of the RTs, LOQs, and the calibration curves are listed in Table S2. After the metabolite profile data were acquired, statistical analysis was performed using MetaboAnalyst 4.0 [40], which is a website for metabolomics data analysis. The Kyoto Encyclopedia of Genes and Genomes (KEGG) database (http://www.genome.jp/kegg) was used for pathways analysis [41].

## 3. Results and Discussion

### 3.1. Separation Parameters of Liquid Chromatography

Methanol was selected as the mobile phase, and the ionization efficiency of the target compounds increased in the positive mode when acetic acid was added. Ultimately, 0.05% acetic acid (*v/v*) was added into the water phase to improve the ionization efficiency in both positive and negative modes. Different sample injection volumes (2, 3, 10 μL) and flow rates (200, 250, 300 μL/min) were also investigated. We selected 3 μL as the optimal injection volume during the analysis. There was no significant difference among flow rates, and 200 μL/min was therefore selected to protect the column from high pressure.

### 3.2. Optimization of the Mass Spectrometry Parameters for Standard Solutions

Mass spectrometric parameters such as precursor ions, product ions, tube lens voltage, and collision energy were optimized by direct injection of each individual analyte (1 μg/mL in methanol) via a syringe pump at a rate of 20 µL/min. Positive and negative modes were both evaluated to optimize precursor ions (positive [M+H]+ and negative [M-H]-). The adduct mode with high intensity for each compound was selected as a precursor ion for the experiment. Then, two product ions of each target compound, with the highest intensities, were selected as the quantitative ion and the qualitative ion, respectively. The optimized SRM scan parameters are described in Table S2.

### 3.3. Oil Sample Analysis and Marker Screening

**Figure 1:**
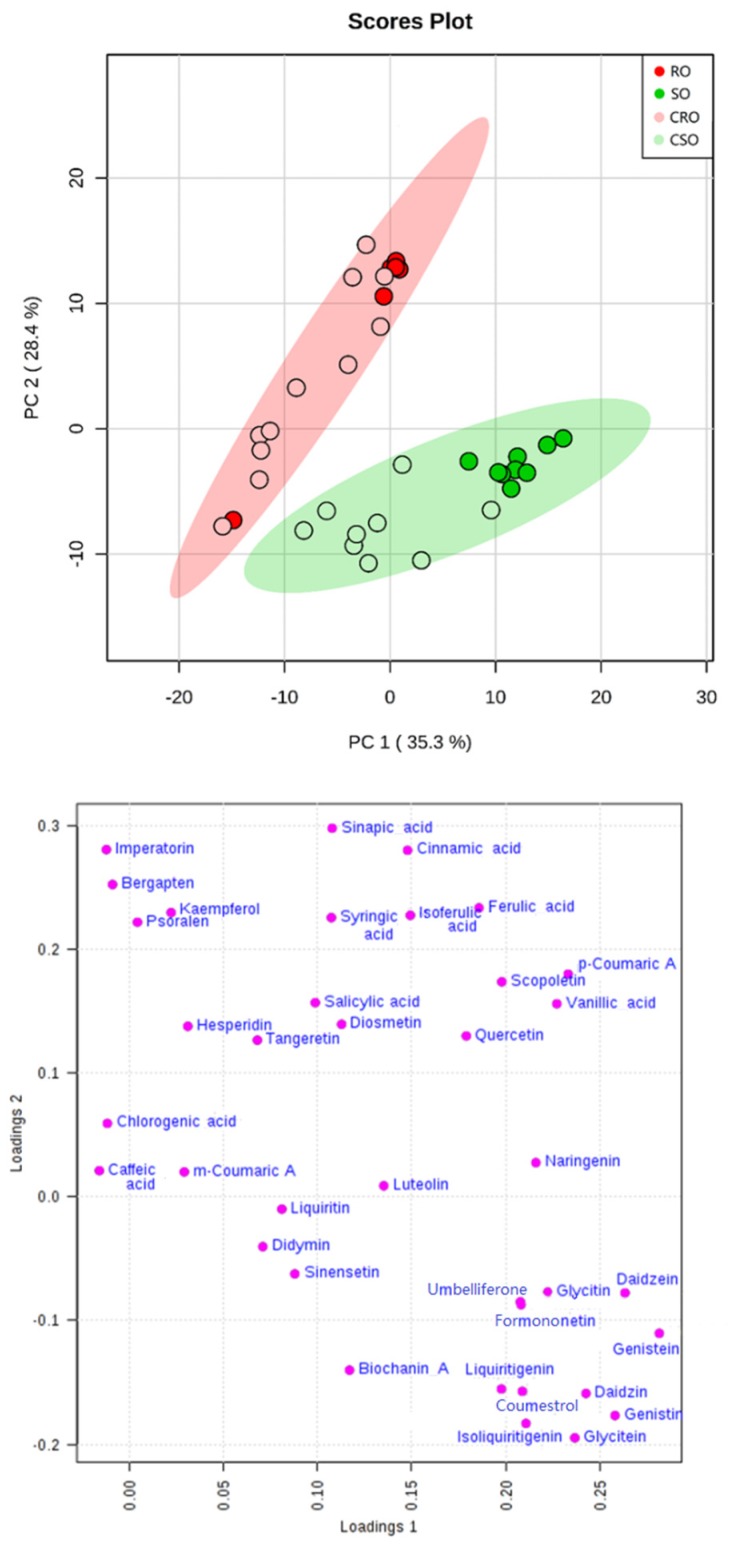
Principal component analysis (PCA) score plot and loading plot of soybean oils and rapeseed oils (RO, Rapeseed oil; CRO, Commercial rapeseed oil; SO, Soybean oil; CSO, Commercial soybean oil).

The analysis of the targets quantified in soybean and rapeseed oils was conducted using the developed method. After normalization by sum and Pareto scaling, principal component analysis (PCA) was performed (Figure 1). The results suggested that the soybean and rapeseed oils could be classified into two distinct categories.

**Figure 2:**
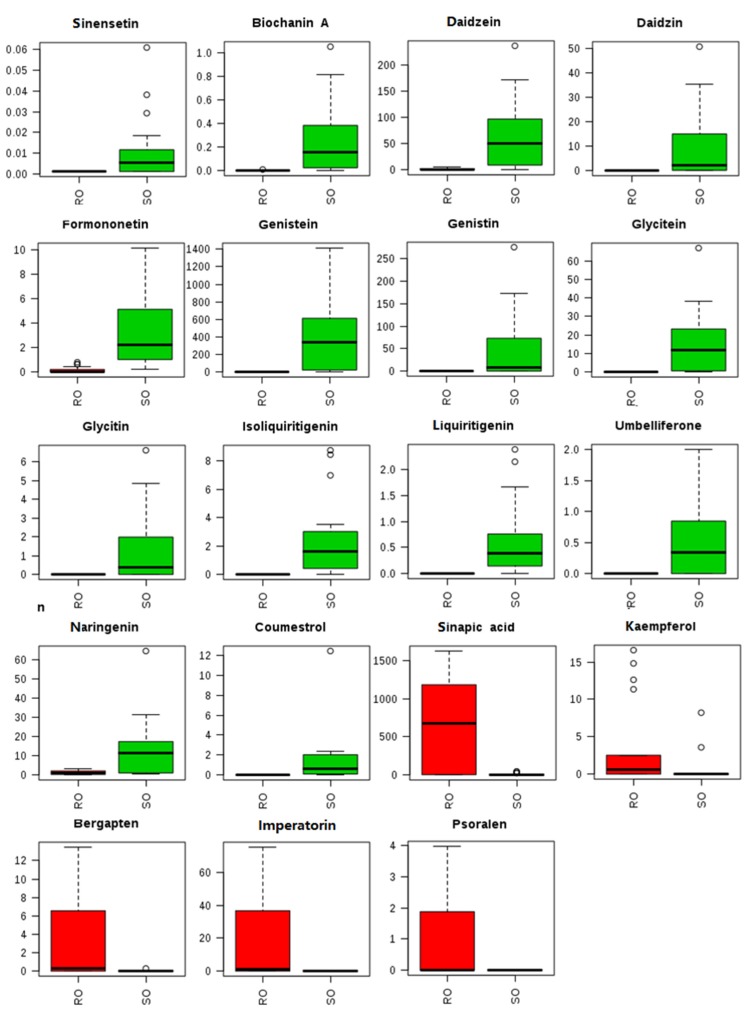
Boxplot of the concentrations of markers in RO and SO (ng/g).

Significantly different metabolites in the soybean and rapeseed oil samples were identified. Figure S1 shows that 19 metabolites showed significantly different abundance (*p* < 0.05). These comprised 14 flavonoids, including isoliquiritigenin, genistin, formononetin, daidzein, liquiritigenin, genistein, coumestrol, glycitein, biochanin A, daidzin, naringenin, glycitin, sinensetin, and umbelliferone, which were found only in soybean oils but not in rapeseed oils. Conversely, five compounds were found at high concentrations in rapeseed oils, including sinapic acid, bergapten, imperatorin, psoralen, and kaempferol, which could be used as marker for rapeseed oils. More importantly, a chain of 11 metabolites in the reference pathway were only present in soybean oils, including daidzein, liquiritigenin, genistein, coumestrol, glycitein, biochanin A, daidzin, naringenin, formononetin, glycitin, and genistin (Figure 2). 

**Figure 3:**
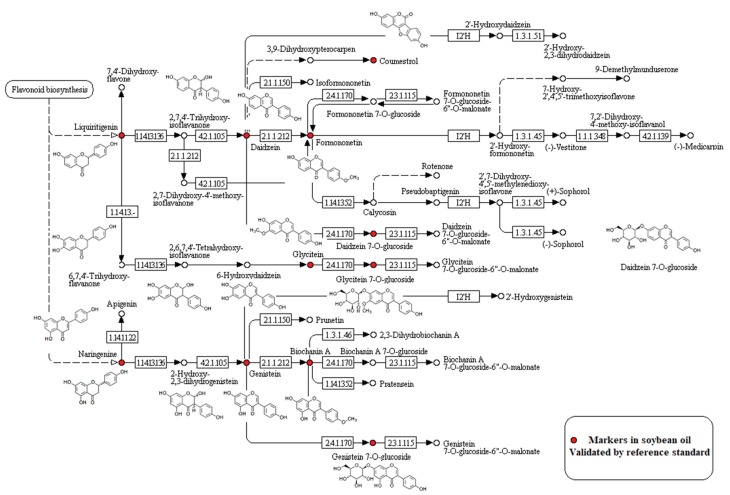
Distribution for metabolic markers of soybean oils in the isoflavonoid biosynthesis pathway, adapted from Kyoto Encyclopedia of Genes and Genomes (KEGG) database [41].

Further analysis indicated that the 11 compounds found at high concentrations in soybean oils were related to the isoflavonoid biosynthesis pathway. Table S3 presents a list of metabolites in the isoflavonoid biosynthesis pathway, of which some were validated by reference standards. Figure 3 shows that there were several metabolites identified as markers of soybean oils. These metabolites, labeled by a red circle, were situated upstream of the isoflavonoid biosynthesis pathway. It can be concluded that these markers are unique to soybean oils and may be used to discriminate soybean from rapeseed oil. These markers are distinct and related to different oil species, rather than as a result of external factors. The results above prove that these differences are caused by genetic heredity, confirming that the markers are species-specific. The false-positive results and false-negative results were significantly reduced by cross-validation, as these markers were simultaneously applied todetect adulterated oils. This method can be used to screen for characteristic markers of other edible vegetable oils.

Compared with previous methods, this method possesses the following advantages: (1) it is based on chains of metabolic markers on the same pathway, which could significantly reduce the occurrence of false-positive results and false-negative results during adulteration detection; (2) it could avoid the identification of unknown metabolites, which remains a big problem in untargeted metabolomics [42,43]; and (3) it could be used directly to detect adulteration or optimized by further selecting highly hydrophobic and thermo-stable markers. Moreover, this method provides a model for screening characteristic markers of other edible vegetable oils and foods.

## 4. Conclusions

Traditional DNA markers are effective in detecting food adulteration of raw materials. However, for processed products such as edible oils, alternative markers are required for more accurate analyses. We developed a targeted metabolomics approach that led to the identification of 56 polyphenols and flavonoids. Liquid/liquid extraction coupled with LC-MS/MS was used to investigate their content in oil samples. Statistical analysis suggested that the markers in soybean oils were concentrated upstream of the isoflavonoid biosynthesis pathway. Ultimately, 11 metabolites overlapped in the isoflavonoid biosynthesis pathway map, which confirmed the effectiveness of marker screening guided by analysis of pathways’ networks. Additionally, the screened markers were used as alternatives to DNA markers to classify soybean and rapeseed oils. The screened markers successfully identified oil species according to their unique pathways’ networks. This method could also be used to screen for other markers of food adulteration in other complex food samples.

## Supplementary Materials

The following are available online at https://www.mdpi.com/2218-1989/10/3/85/s1, Figure S1: T-test plot of significant differences between the variables, Table S1: Detailed information of the commercial oil samples used, Table S2: Retention times, scan parameters, and calibration curves of the target compounds, Table S3: Observation of metabolites in the isoflavonoids biosynthesis pathway.
