# Supplementary material for: Identification and Validation of Metabolic Markers for Adulteration Detection of Edible Oils Using Metabolic Networks
Source: Metabolites. 2020 Feb 29;10(3):85. doi: 10.3390/metabo10030085 (PMC7143555; doi:10.3390/metabo10030085)
Supplement: Supplementary file 1 [file metabolites-10-00085-s001.pdf]

Supplementary materials:

Figure S1 T-test plot of the significant difference in variables

Table S1 Detail information of commercial oil samples

Table S2 Retention time, scan parameters and calibration curve of target compounds

Table S3 Observation of metabolites in isoflavonoids biosynthesis pathway

Figure S1 T-test plot of the significant difference in variables

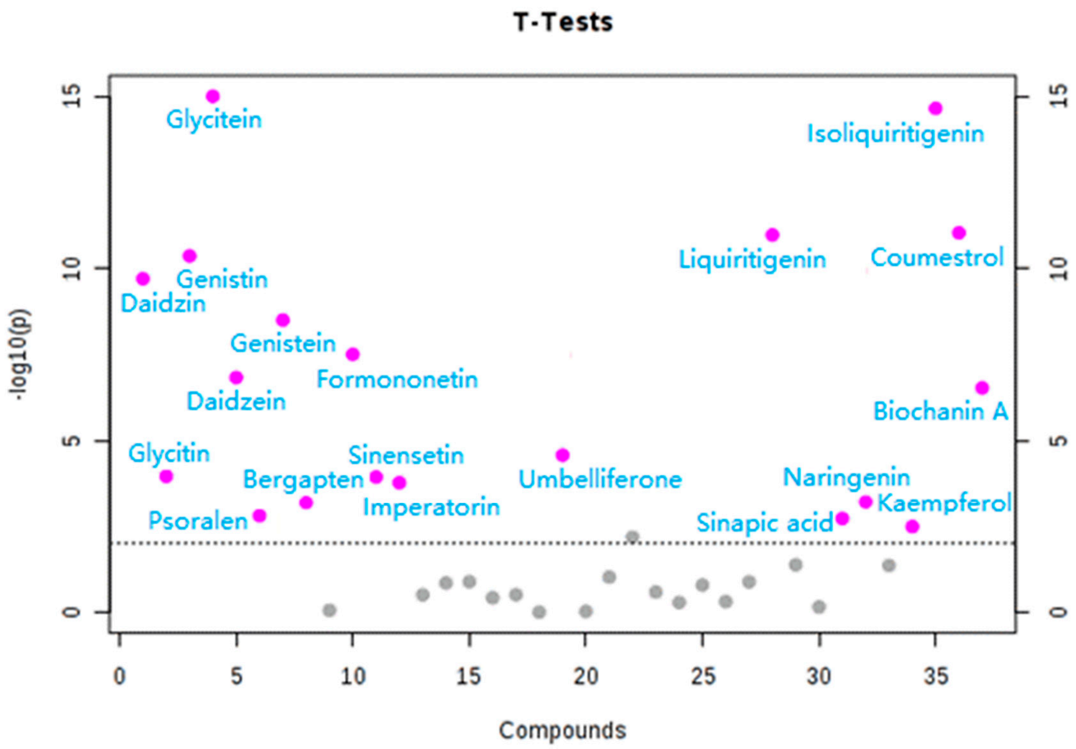

Table S1 Detail information of commercial oil samples

Sample No.	Vendor	Origin of raw materials	Supermarket	Specifications
CSO1	Lin Long	Wuhan, Hubei	Carrefour, Wuhan	5L
CSO2	Jin Longyu	Wuhan, Hubei	Supermarket, Wuhan	1.8L
CSO3	Fu Linmen	Suzhou, Jiangsu	RT-MART, Wuhan	1.8L
CSO4	Fu Linmen	Tianjin	Jingkelong Supermarket, Beijing	900mL
CSO5	Zhong An	Heilongjiang	WU MART, Beijing	5L
CSO6	Hong Qingting	Chongqing	Yonghui Superstore, Chongqing	5L
CSO7	Ying Mai	Zhongshan, Guangdong	Supermarket, Chongqing	5L
CSO8	Yuan Bao	Guangzhou, Guangdong	Carrefour, Guangzhou	5L
CSO9	Jin Ye	Zhenjiang, Jiangsu	Supermarket, Hangzhou	1.8L
CRO1	Dao Daoquan	Nanjing, Jiangsu	RT-MART, Wuhan	1.8L
CRO2	Ao Xing	Xiangyang, Hubei	RT-MART, Wuhan	1.8L
CRO3	Hengda Xing'an	Huhehot, Inner Mongolia	Jingkelong Supermarket, Beijing	500mL
CRO4	Xian Can	Chengdu, Sichuan	Supermarket, Chongqing	900mL
CRO5	Hong Qingting	Chongqing	Yonghui Superstores, Chongqing	1L
CRO6	Wu Hu	Huanggang, Hubei	Supermarket, Chongqing	5L
CRO7	Dao Mai	Shenzhen, Guangdong	Supermarket, Guangzhou	2L
CRO8	Lao Xiang	Shenzhen, Guangdong	Carrefour, Guangzhou	900mL
CRO9	Fu Linmen	Maoming, Guangdong	Supermarket, Chongqing	900mL
CRO10	Fu Linmen	Suzhou, Jiangsu	Supermarket, Hangzhou	1.5L
CRO11	Chu Laixiang	Hangzhou, Zhejiang	Supermarket, Hangzhou	1.8L

CSO, Commercial soybean oil   CRO, Commercial rapeseed oil

Table S2 Retention time, scan parameters and calibration curve of target compounds

No.	Compound	Scan mode	RT min	Precursor ion (m/z)	Daughter ion[1]*(m/z)	CE[1] (eV)	Daughter ion[2](m/z)	CE[2] eV	Tube lens(V)	Calibration curve	R^2	LOQ （ng/mL）	Liner range (ng/mL)
1	Catechinic	-	5.16	289	245	15	139	30	120	Y = 211296+725.28*X	0.9329	1.05	1.05-800
2	Scopolin	+	5.38	355	193	16	133	34	68	Y = 925678+66176*X	0.9910	0.35	0.35-1000
3	Chlorogenic acid	-	5.38	353	191	21			100	Y = -313569+15919.2*X	0.9838	0.16	0.16-2000
4	Epicatechinic	-	5.82	289	245	15	139	30	120	Y = 78580.8+1044.04*X	0.9893	5.23	5.23-2000
5	Vanillic acid	-	5.84	167	108	21	123	15	105	Y = 1874.95*X	0.9880	3.42	3.42-2000
6	Caffeic acid	-	5.88	179	135	18	119	18	100	Y = 9695.33*X	0.9901	1.52	1.52-2000
7	Purerarin	-	5.91	415	267	36	295	24	65	Y = 196697+64919.7*X	0.9937	0.10	0.10-2000
8	Syringic acid	-	6.03	197	182	15	123	26	63	Y = 5856.18*X	0.9823	1.73	1.73-1000
9	Daidzin	+	6.28	417	199	45	255	22	160	Y = 207244*X	0.9932	0.36	0.36-1000
10	Glycitin	+	6.38	447	285	22	270	38	102	Y = 179646*X	0.9959	0.28	0.28-2000
11	Scopoletin	-	6.6	191	176	16	148	22	48	Y = 21877.4*X	0.9952	0.54	0.36-2000
12	Eriocitrin	-	6.63	595.5	287	22	151	38	105	Y = 14784*X	0.9974	0.11	0.11-800
13	Umbelliferone	-	6.67	161	133	21	105	25	143	Y = 8475.21*X	0.9918	0.25	0.25-2000
14	p-Coumaric Acid	-	6.69	163	119	15			67	Y = 14151.8*X	0.9940	10.0	10-2000
15	Dihydroquercetin	-	6.76	303	285	13	125	22	75	Y = 18901.7*X	0.9921	1.22	1.22-1500
16	Sinapic acid	-	6.77	223	208	17	193	25	136	Y = 8859.22*X	0.9960	0.88	0.88-800
17	Genistin	+	6.8	433	271	27			127	Y = 95819.4*X	0.9960	0.15	0.15-1500
18	Liquiritin	-	6.84	417	255	19	135	32	77	Y = 34023.3*X	0.9925	0.10	0.10-2000
19	Ferulic acid	-	6.84	193	134	18	178	16	57	Y = 12965.5*X	0.9947	1.93	1.93-1000
20	Salicylic acid	-	6.98	137	93	25	106	19	100	Y = 15352.8*X	0.9954	1.24	1.24-2000

21	Rutin	+	7.02	611	303	22	465	12	100	Y = 127601*X	0.9942	0.02	0.02-2000
22	Isoferulic acid	-	7.03	193	134	18	178	16	57	Y = 13517.5*X	0.9914	0.97	0.97-1000
23	m-Coumaric Acid	-	7.17	163	119	15			67	Y = 98331.7*X	0.9779	2.78	2.78-500
24	Naringin	-	7.2	579.5	271	26	151	43	110	Y = 15914.3*X	0.9920	0.10	0.10-2000
25	Hesperidin	-	7.27	609.5	301	26			81	Y = -110616+13574.9*X	0.9985	0.12	0.12-2000
26	Resveratrol	-	7.32	227	185	22	143	20	137	Y = 13238.6+4202.5*X	0.9945	0.84	0.84-1000
27	Xanthotoxol	+	7.42	203	147	23	91	33	101	Y = 329317+100504*X	0.9898	1.91	1.91-800
28	Silydianin	-	7.55	481	151	30	453	21	90	Y = -136398+7253.64*X	0.9979	0.33	0.33-2000
29	Sinapyl alcohol	-	7.69	209	119	20			59	Y = -250416+21135.1*X	0.9961	1.47	1.47-800
30	o-Coumaric Acid	-	7.69	163	119	15			67	Y = 78681.8*X	0.9963	0.91	0.91-1000
31	Liquiritigenin	-	7.95	255	119	23	135	17	57	Y = 32905.3*X	0.9976	0.74	0.74-800
32	Kaempferol	-	7.96	285	185	28	117	43	90	Y = 40365.9+9099.44*X	0.9763	2.73	2.73-500
33	2'-Hydroxygenistein	-	7.96	285	217	21	175	26	80	Y = 26973.7*X	0.9914	0.98	0.98-2000
34	Eriodictyol	-	8.03	287	151	16	135	27	65	Y = 21770.1+22372*X	0.9923	1.38	1.38-1000
35	Daidzein	+	8.15	255	137	26	199	30	132	Y = 83185.3*X	0.9929	0.17	0.17-800
36	Psoralen	+	8.34	187	131	23	77	36	76	Y = 146787*X	0.9826	1.21	1.21-800
37	Glycitein	+	8.35	285	270	25	242	30	93	Y = 279506*X	0.9951	0.07	0.07-800
38	Quercetin	-	8.36	301	151	23	179	20	140	Y = -71762.3+11903.2*X	0.9965	3.05	3.05-2000
39	Didymin	-	8.42	593.5	285	30			121	Y = 12723.5*X	0.9936	0.03	0.03-1000
40	Bergaptol	+	8.55	203	147	22	131	21	100	Y = 217614*X	0.9903	1.08	1.08-500
41	Naringenin	+	8.66	273	153	24	147	19	84	Y = 26591*X	0.9913	1.23	1.23-800
42	Luteolin	-	8.66	285	133	39			134	Y = 15309*X	0.9971	2.60	2.60-2000
43	Cinnamic_acid	-	8.79	147	103	14			52	Y = -1048.37+1744.31*X	0.9967	0.21	0.21-2000
44	Hesperetin	+	8.85	303	153	22	177	17	81	Y = 113186*X	0.9969	0.60	0.6-500
45	Genistein	+	8.86	271	153	27	215	25	127	Y = 69082.5*X	0.9938	0.43	0.43-800
46	Bergapten	+	9.26	217	202	20	90	37	102	Y = -818529+397419*X	0.9972	0.20	0.20-800

47	Diosmetin	+	9.49	301	286	25	258	33	87	Y = 195907*X	0.9984	0.58	0.58-1000
48	Isoliquiritigenin	-	9.51	255	119	27	135	17	72	Y = 56904.2*X	0.9932	1.79	1.79-1500
49	Coumestrol	-	9.51	267	266	27	211	29	180	Y = 27834.9*X	0.9938	0.53	0.53-2000
50	Sinensetin	+	9.92	373	343	27	312	23	98	Y = 532301*X	0.9919	0.16	0.16-1000
51	Formononetin	-	9.93	267	252	21	223	34	200	Y = 139585*X	0.9944	0.41	0.41-1000
52	Medicarpin	-	10.15	269	254	19	211	33	90	Y = 21889*X	0.9925	1.30	1.30-1000
53	Imperatorin	+	10.99	271	203	21	147	31	63	Y = 236190*X	0.9967	1.16	1.16-2000
54	Biochanin A	-	11	283	268	22	239	32	78	Y = 129780*X	0.9947	0.66	0.66-1000
55	Tangeretin	+	11.76	373	343	24	312	23	94	Y = 838308*X	0.9948	0.15	0.15-1500
56	Rotenone	+	11.97	395	213	22	192	19	99	Y = 172294*X	0.9945	0.22	0.22-1000

* Quantitative ion，CE，Collision energy

Table S3 Observation of metabolites in isoflavonoid biosynthesis pathway

No.	KEGG ID	Compound	Formula	CAS No.	Structural Formula
1	C09762	Liquiritigenin	C15H12O4	578-86-9	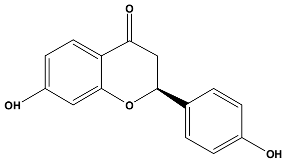
2	C10208	Daidzein	C15H10O4	486-66-8	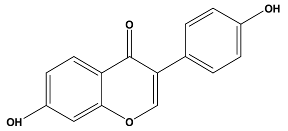
3	C10216	Daidzein 7-O-glucoside	C21H20O9	552-66-9	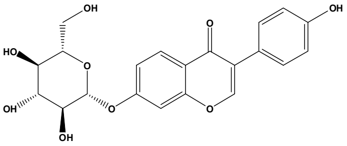
4	C10205	Coumestrol	C15H8O5	479-13-0	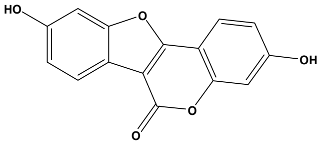
5	C00858	Formononetin	C16H12O4	485-72-3	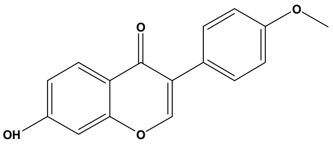
6	C14536	Glycitein	C16H12O5	40957-83-3	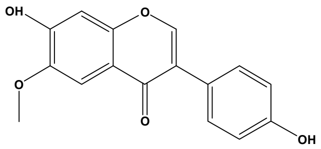
7	C16195	Glycitin	C22H22O10	40246-10-4	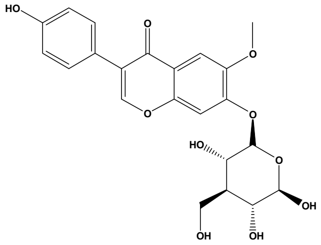
8	C00509	Naringenin	C15H12O5	480-41-1	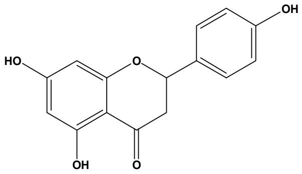
9	C06563	Genistein	C15H10O5	446-72-0	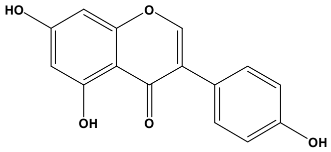

10	C00814	Biochanin A	C16H12O5	491-80-5	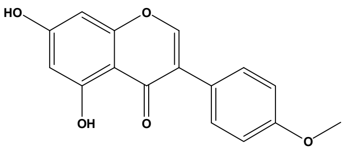
11	C09126	Genistein 7-O-beta-D-glucoside	C21H20O10	529-59-9	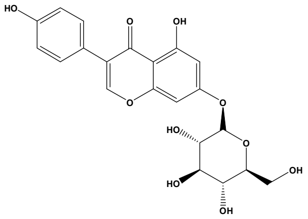
